# Recombinant expression and characterization of the endochitinase Chit36-TA from *Trichoderma asperellum* in *Komagataella phaffii* for chitin degradation of black soldier fly exuviae

**DOI:** 10.1007/s00449-024-03067-4

**Published:** 2024-08-08

**Authors:** Luisa Gebele, Andreas Wilke, Axel Salliou, Laura Schneider, Daniel Heid, Tobias Stadelmann, Corinna Henninger, Uzair Ahmed, Melanie Broszat, Pascale Müller, Georg Dusel, Michał Krzyżaniak, Katrin Ochsenreither, Thomas Eisele

**Affiliations:** 1 Faculty of Mechanical and Process Engineering, Hochschule Offenburg, 77652 Offenburg, Germany; 2 École Supérieure de Biotechnologie de Strasbourg, 67412 Illkirch Cedex, France; 3 Department Life Sciences and Engineering, Technische Hochschule Bingen, 55411 Bingen am Rhein, Germany; 4 Department of Genetics, Plant Breeding and Bioresource Engineering, University of Warmia and Mazury in Olsztyn, Plac Łódzki 3, 10-724 Olsztyn, Poland; 5 Department of Chemical and Process Engineering, Karlsruhe Institute of Technology (KIT), 76131 Karlsruhe, Germany

**Keywords:** Chitinases, Biopolymer, Chitin, *Komagataella phaffii*, *Trichoderma asperellum*, Black soldier fly larvae

## Abstract

The natural polymer chitin is an abundant source for valuable N-acetylchitooligosaccharides and N-acetylglucosamine applicable in several industries. The endochitinase Chit36-TA from *Trichoderma asperellum* was recombinantly expressed in *Komagataella phaffii* for the enzymatic degradation of chitin from unused insect exuviae into N-acetylchitooligosaccharides. Chit36-TA was purified by Ni–NTA affinity chromatography and subsequently biochemically characterized. After deglycosylation, the endochitinase had a molecular weight of 36 kDa. The optimum pH for Chit36-TA was 4.5. The temperature maximum of Chit36-TA was determined to be 50 °C, while it maintained > 93% activity up to 60 °C. The chitinase was thermostable up to 45 °C and exhibited ~ 50% activity after a 15 min incubation at 57 °C. Chit36-TA had a maximum specific enzyme activity of 50 nkat/mg with a K_m_ value of 289 µM with 4-methylumbelliferyl-N,N′,N″-triacetyl-*β*-chitotrioside as substrate. Most tested cations, organic solvents and reagents were well-tolerated by the endochitinase, except for SDS (1 mM), Cu^2+^ (10 mM) and Mn^2+^ (10 mM), which had stronger inhibitory effects with residual activities of 3, 41 and 28%, respectively. With a degree of hydrolysis of 32% applying colloidal shrimp chitin (1% (w/v)) and 12% on insect larvae (1% (w/v)) after 24 h, the endochitinase was found to be suitable for the conversion of colloidal chitin as well as chitin from black soldier fly larvae into water-soluble N-acetylchitooligosaccharides. To prove scalability, a bioreactor process was developed in which a 55-fold higher enzyme activity of 49 µkat/l and a tenfold higher protein expression of 1258 mg/l were achieved.

**Supplementary Information:**

The online version contains supplementary material available at 10.1007/s00449-024-03067-4.

## Introduction

Chitin is a natural biopolymer with an estimated annual production of 10^11^ tons, it is the second most abundant biomolecule on earth after cellulose [1]. Its structure consists of several hundreds to thousands of *β*-(1,4)-O-glycosidic-linked N-acetylglucosamine (GlcNAc) units [2]. Chitin is water insoluble [3] and due to this property, the application of the raw material is often limited. The hydrolytic products, so-called N-acetylchitooligosaccharides or the monomer N-acetylglucosamine, are water soluble due to their shorter polymer chain length. In addition, the solubility depends on the polymerization degree and decrease significantly for oligomers with higher polymer chain lengths (*n* ≥ 5) [4]. Both have a wider range of application [5] in industrial sectors such as agriculture [6], cosmetics [7], food [8] and medicine [9].

Chitin has been discovered in a wide variety of organisms as a major cell wall component of many fungi, crustacean shells as well as shellfish and insect exoskeletons and exuviae [5]. As the world’s population continues to increase to an estimated 9.4 to 10.1 billion people by 2050 [10], the need to find alternatives of high-value protein sources is an urgent matter. In addition to the traditional diet of food crops and livestock, sufficient alternative sources must be found. Insects such as the black soldier fly (*Hermetia illucens*) are receiving increasing attention in the food industry as novel protein sources due to their high protein content [1, 11]. The global market for edible insects, which are produced in insect farms, is estimated at USD 8 billion in 2030 [12], which is a significant increase on the initial estimate of USD 522 million in 2023 [1]. In addition, European insect food business operators produced around 500 t of insect-based products for sale on the European market in 2019. It is anticipated that 260 kt of insect-based products will be required by 2030 to meet the increasing demand only in Europe [13].

During insect farming, insects such as the black soldier fly release several larval exoskeletons and pupal exuviae within the different developmental stages (egg, larva (maggot), prepupa, pupa and adult fly) [14]. As a result, insect exuviae and exoskeletons are produced as unused byproduct streams. These new cheap sources of chitin, which consist of 2 to 23% chitin [1, 15, 16], depending on the origin and growth phases of the insect and the applied analytical method, can be made usable by degrading chitin. The insect exuviae can be compared with the shells of crustaceans from the fishing industry in terms of components (minerals, proteins and chitin) and treatment for chitin extraction. The major difference is the composition. Crustaceans have more minerals such as calcium, phosphorus, potassium and magnesium salts and in average a higher chitin content [5]. The marine food industry is currently still the largest source of chitin with 8 million tons of crustaceans reported in 2016, whereas 40% of the amount consisting of chitinous exoskeletons. However, in view of the sustainability aspect and the expected increase in insect production, this could change and byproducts like larval exoskeletons and pupal exuviae represent a more interesting source of chitin for the future [1, 17]

Currently, chemical depolymerization deploying mostly highly concentrated hydrochloric acid and high temperatures is applied for oligomer production from insect exuviae [1, 18]. Due to the use of strong acids, as well as the high energy expenditure, this process is not environmentally friendly [19]. A more environmentally friendly alternative is enzymatic depolymerization using chitinases. These glycosidases catalyze the hydrolytic cleavage of the *β*-(1,4)-O-glycosidic bonds between N-acetylglucosamine units in chitin [20]. Chitinases can be further classified as either endochitinases or exochitinases. Endochitinases (EC 3.2.1.14) cleave chitin at random internal O-glycosidic bonds and produce soluble products of low molecular weight, such as tetraacetylchitotetraose, triacetylchitotriose and diacetylchitobiose [21]. Exochitinases catalyze the release of diacetylchitobiose and GlcNAc and include chitobiosidases and *β*-(1,4)-N-acetylglucosaminidases (EC 3.2.1.52). Chitobiosidases act on the non-reducing regions of chitin and produce dimers from the main chitin chain that can be converted to sugar monomers by *β*-(1,4)-N-acetylglucosaminidases [2]. However, the biotechnological processes developed so far have been associated with lower yields and high time requirements [1]. Furthermore, chitinases are not readily available commercially or, if available, are very expensive [19].

Therefore, the aim of this work was to produce a recombinant endochitinase on a larger scale, to analyze its biochemical characteristics and evaluate its ability to degrade chitin directly from insect exuviae to develop an efficient biotechnological method for chitin valorization. To select an appropriate candidate, chitinases from entomopathogenic fungi as well as chitinase genes from other fungi that are potentially able to hydrolyze the chitin from insect cuticles were selected and studied. The ideal candidate identified as Chit36-TA from *Trichoderma asperellum* CBS 433.97 should have appropriate biochemical properties such as broad pH range and high temperature stability as well as high tolerance against cations, solvents and reagents for industrial use. To the best of our knowledge, it has been shown for the first time that the endochitinase Chit36-TA hydrolyzes the chitin from non-pretreated exuviae from black soldier fly larvae and thus may be suitable for alternative N-acetylchitooligosaccharide production.

## Materials and methods

### Chemicals and equipment

All chemicals and reagents were of analytical grade and were obtained from either Carl Roth (Karlsruhe, Germany), Th. Geyer (Renningen, Germany) or Merck (Darmstadt, Germany), unless stated otherwise. The substrate 4-methylumbelliferyl-N,N′,N″-triacetyl-*β*-chitotrioside (4-MuChT), the standard 4-methyl-umbelliferone (4-MU) and N,N′-diacetylchitobiose, were obtained from Merck (Darmstadt, Germany), whereas the α-chitin from shrimp shells and N-acetyl-D-glucosamine were purchased from Carl Roth (Karlsruhe, Germany). N,N',N''-triacetylchitotriose was ordered from Cayman Chemicals (Michigan, USA) and N,N',N'',N''',N''''-pentaacetylchitopentaose was obtained from Biosynth (Staad, Switzerland). Peptide-N-Glycosidase F (PNGase F) was ordered from New England Biolabs (Frankfurt am Main, Germany), and bovine serum albumin (BSA) was obtained from Thermo Fisher Scientific (Dreieich, Germany).

### *Komagataella phaffii* transformation

The native protein sequence without the original signal peptide of *Trichoderma asperellum*´s endochitinase (UniProt accession A0A2T3ZD30) was back-translated and codon optimized for expression *in Komagataella phaffii* using the Geneious software (Dotmatics, Boston, USA) and the corresponding algorithm for codon optimization. The synthetic gene with a C-terminal hexa-histidine tag (Supplementary material ESM 1) was ordered as a clonal gene fragment in the expression plasmid pBSY2S1Z (Bisy, GmbH, Graz, Austria) from Twist Bioscience (San Francisco, CA, USA). The expression host, *Komagataella phaffii* BG10, was obtained from Bisy GmbH (Graz, Austria). The pBSY2S1Z_Chit36-TA plasmid (Supplementary material ESM 2) was linearized using Sac I HF (NEB, Frankfurt am Main, Germany) according to the manufacturer’s instructions. For the linearization reaction, 1–3 µg of plasmid DNA, 3 µl of rCutSmart™ Buffer (NEB, Frankfurt am Main), and 1 µl of Sac I HF were incubated in a total volume of 30 µl (distilled H_2_O) for 30 min at 37 °C in a PCR tube. Subsequently, the linearized DNA was purified using the ZYMO RESEARCH DNA Clean & Concentrator Kit-5 (Zymo Research, Freiburg im Breisgau, Germany). The linearized and purified DNA fragment was used to transform chemically competent *Komagataella phaffii* BG10 (*K. phaffii*) cells. These competent cells were prepared and transformed using the Frozen-EZ Yeast Transformation II™ Kit (Zymo Research, Freiburg im Breisgau, Germany) following the manufacturer’s protocol. *K. phaffii* cells were grown in yeast extract peptone dextrose (YPD) medium (g/l: yeast extract 10, peptone 20, glucose 20) to an OD_600nm_ of 0.8–1.0 in the mid-log phase. The cells were then centrifuged at 500 × g for 4 min, and the supernatant was discarded. The pellet was washed with 10 ml of Frozen-EZ Yeast Solution 1, centrifuged again at 500 × g for 4 min, and the supernatant was discarded. Finally, the pellet was resuspended in 1 ml of Frozen-EZ Yeast Solution 2. The resulting competent cells were either used for transformation or stored at −70 °C for future use. The transformed cells were selected on YPD plates (g/l: yeast extract 10, peptone 20, glucose 20 and agar–agar 20) containing 100 µg/ml Zeocin^®^ (InvivoGen, Toulouse, France). The YPD-Zeocin agar plates were incubated at 30 °C for 3 days. After incubation, three colonies were picked for test expression in a 24-well microplate at a 1.0 ml scale using BMGY media containing 100 µg/ml Zeocin. The microplate was incubated at 30 °C in a shaker at 120 rpm (Kelvin + , Kuhner, Birsfelden, Switzerland) for 24 h. Following this incubation, 100 µl of the *K. phaffii* cell suspension was transferred into 900 µl of buffered methanol complex (BMMY) medium (g/l: yeast extract 10, peptone 20, yeast nitrogen base 13.4, biotin 0.4∙10^–3^, methanol 0.5% (v/v) and potassium phosphate 100 mM pH 6.5) to induce the tightly regulated and strictly methanol-inducible AOX1 promoter [22]. The microplate was incubated at 30 °C and 120 rpm for an additional 72 h, with 0.5% (v/v) methanol being added after 24 h and 48 h.

### Recombinant expression of Chit36-TA in shake flasks

For protein expression, *Komagataella phaffii* transformants were grown on YPD plates and incubated at 30 °C for three to four days to isolate single colonies. Endochitinase production was carried out in a two-stage procedure with a preculture and a main culture, both cultures using buffered glycerol complex (BMGY) medium (g/l: yeast extract 10, peptone 20, yeast nitrogen base 13.4, biotin 0.4∙10^–3^, glycerol 2.0% (w/v) and potassium phosphate 100 mM pH 6.5). For preculture, a single colony was inoculated in 20 ml BMGY medium in a 100 ml shake flask at 28 °C at 140 rpm for 24 h. The main culture with a working volume of 400 ml in a 2 l shake flask was inoculated with 20 ml of the preculture and shaken at 28 °C at 110 rpm for two days. Next, 0.5% (v/v) methanol was added to induce protein expression. Methanol addition (0.5% (v/v)) was performed every 24 h. After 72 h, the yeast cells were harvested by centrifugation at 13,400 × g at 4 °C for 10 min and the supernatant including the recombinantly expressed and secreted endochitinase was sterile filtered (0.22 µm, TTP®, Trasadingen, Switzerland). OD_600nm_, enzyme activity and protein concentration were determined during shake flask cultivation. When measuring the protein concentration, the protein content in the complex medium (BMGY) was substracted to display only the relevant enzyme concentration. For further observation under a scanning electron microscope, samples were taken from the cell pellets and frozen at - 80 °C until use.

### Purification of Chit36-TA

After sterile filtration (0.22 µm) of the supernatant, the endochitinase Chit36-TA was purified by Ni–NTA chromatography on an ÄKTA Go with a HiTrap™ IMAC HP 5 ml column (GE Healthcare, Uppsala, Sweden). A solution of 300 mM NaCl, 2 mM imidazole and 50 mM sodium phosphate buffer (pH 7.4) served as the binding buffer. The elution buffer consisted of 300 mM NaCl, 500 mM imidazole and 50 mM sodium phosphate buffer (pH 7.4). Before loading the column, the filtered supernatant was prepared in the binding buffer and cooled on ice during chromatography. For purification, a flow rate of 2 ml/min was set at a maximum pressure of 0.5 MPa. After equilibration (5 column volume, CV) and sample loading, washing was performed with 8 CV using binding buffer. This was followed by a step elution with 100% elution buffer. Purified protein was dialyzed against 5 mM sodium acetate buffer (pH 5.0) with a Nadir^®^ dialysis tube (10—20 kDa, Carl Roth, Karlsruhe, Germany).

### Protein quantification by Bradford-assay

The protein content was quantified using the Bradford assay [23] in a microtiter plate (Carl Roth, Karlsruhe, Germany). The Bradford solution ROTI^®^ Nanoquant 5x (Carl Roth, Karlsruhe, Germany) was used to measure the protein concentration in the culture supernatant as well as in the purified samples. The ratio of OD_595 nm_/OD_450 nm_ was determined according to the manufacturer's instructions and converted to a protein concentration using a calibration curve with a bovine serum albumin (BSA) standard. The spectrophotometric measurements were performed in an Epoch 2 Micro Plate Reader manufactured by Biotek (Winooski, VT, USA).

### Deglycosylation reaction with PNGase F

The purified Chit36-TA was deglycosylated using the PNGase F enzyme from New England Biolabs (Frankfurt am Main, Germany), according to the manufacturer’s instructions for the denaturing method with slight modifications. The sample was denatured at 95 °C for 5 min, in contrast to the manufacturer’s protocol. The reaction mixture consisting of 10 µg of Chit36-TA was incubated using the ThermoMixer^®^ C (Eppendorf, Hamburg, Germany) for 14 h at 37 °C under gentle shaking.

### Sodium dodecyl sulfate polyacrylamide gel electrophoresis and molecular mass determination

Sodium dodecyl sulfate–polyacrylamide gel electrophoresis was performed using a modified Laemmli protocol [24]. A 4–20% Novex™ WedgeWell™ Tris–Glycine Gel, (Invitrogen, Thermo Fisher Scientific, Dreieich, Germany) was used for protein separation. ROTI^®^ Load 1 4X (Carl Roth, Karlsruhe, Germany) was used as a protein gel loading buffer. All the samples were appropriately diluted and heated at 95 °C for 5 min. The protein marker PageRuler Unstained Protein Ladder (Thermo Fisher Scientific, Dreieich, Germany) was used as a reference for the molecular mass calculations. The samples were separated using Laemmli running buffer (SERVA Electrophoresis GmbH, Heidelberg, Germany) at a constant voltage of 200 V in a Mini Gel Tank (Thermo Fisher Scientific, Dreieich, Germany). The protein bands on the gel were visualized using GelCode™ Blue Safe Protein Stain staining solution (Thermo Fisher Scientific, Dreieich, Germany) and digitized using the GelDoc Go Imaging System (Bio-Rad, Hercules, CA, USA).

### Preparation of colloidal chitin

The modified method of Songsiriritthigul et al. [25] was used to prepare colloidal chitin. In this method, 10 g of commercial chitin from shrimps was slowly added to 250 ml of concentrated hydrochloric acid (32% (w/w)). The suspension was stirred for 2 h at room temperature. This was followed by overnight incubation at 4 °C. The solution was slowly added to 50% (w/v) ice-cold ethanol and stirred for 10 min. The white precipitate was filtered by a cotton cloth using a Büchner funnel and a suction bottle (Carl Roth, Karlsruhe, Germany). The colloidal chitin mixture was washed with distilled water until a pH of 6 was reached. From the chitin suspension, 1 g was used to determine the dry mass using a Kern DBS moisture analyzer (Kern & Sohn, GmbH, Balingen, Germany). For the determination of enzyme activity using dinitrosalicylic acid assay, a 1% (w_dry_/v) suspension was prepared using 50 mM sodium acetate buffer pH 4.5 and stored at 4 °C.

### Determination of endochitinase activity with 4-methylumbelliferyl-N,N′,N″-triacetyl-*β*-chitotrioside (4-MUChT) assay

The 4-MUChT assay with 4-methylumbelliferyl-N,N′,N″-triacetyl-*β*-chitotrioside was carried out according to modified methods of Pérez-Martínez et al. [26] and McCreath and Gooday [27]. The activity of the endochitinase was determined by preincubating 30 µl of appropriately diluted enzyme solution in 70 µl of 50 mM acetate buffer pH 4.5 for 2 min at 40 °C. After adding 20 µl of 120 µM 4-MUChT, the mixture was incubated at 40 °C under shaking for 5 min (standard assay conditions), then terminated by adding 120 µl of 500 mM sodium carbonate. The total volume of the 4-MUChT assay was 240 µl. Fluorescence was quantified on a CLARIOstar^®^ Plus Plate Reader (BMG LABTECH, Ortenberg, Germany) at 365 nm excitation and 445 nm emission wavelengths. For calibration, a 4-methylumbelliferone (4-MU) standard was used to convert the relative fluorescence unit (RFU) to a molar concentration. One nanokatal (nkat) or one microkatal (µkat) of endochitinase activity was defined as the release of 1 nmol or 1 µmol of 4-MU equivalents per second. Here, 1 kat corresponds to 6∙10^7^ units (U).

## Biochemical characterization of Chit36-TA

All measurements of the biochemical characterization were performed by employing the fluorometric 4-MUChT assay in duplicates. The determination of effects of cations, solvents and reagents on endochitinase activity was performed in four replicates.

### Determination of the pH optimum, temperature maximum and temperature stability

To study the pH dependency of endochitinase activity, different buffers (50 mM) from pH 3.5 to 9.0 were used (pH 3.5–5.0 sodium acetate; pH 6.0–7.0 sodium phosphate; pH 8.0–9.0 Tris–HCl). The temperature optimum of the chitinase was determined using the 4-MUChT assay with varying incubation temperatures from 30 to 90 °C. To determine the thermal stability, the enzyme activity was measured after incubating the enzyme solution at 40 to 75 °C for 15 min with subsequent cooling on ice.

### Determination of kinetic parameters

The kinetic parameters V_max_ and K_m_ of Chit36-TA were determined using final substrate concentrations ranging from 5 to 70 µM 4-MUChT at 40 °C and pH 4.5. The parameters were calculated using SigmaPlot 15.0 by employing the linearization methods Lineweaver–Burk and Hanes-Woolf [28] and a nonlinear regression.

### Effects of cations, solvents and reagents on endochitinase activity

The effects of several cations, solvents and reagents on chitinase activity were analyzed. Chitinase activity was assayed by reaction mixtures consisting of chloride salts of Na^+^, K^+^, Ca^2+^, Cu^2+^, Co^2+^, Mg^2+^, Mn^2+^ and Zn^2+^ and the reagents SDS (sodium dodecyl sulfate), EDTA (ethylenediaminetetraacetic acid), urea, DDT (dichlorodiphenyltrichloroethane) and *β*-mercaptoethanol at final concentrations of 0.1, 1 and 10 mM. The solvents DMSO (dimethyl sulfoxide), ethanol, acetone and DMF (dimethylformamide) were added at a final dilution of 1:10. Subsequently, enzyme activity was measured at pH 4.5 and 40 °C after preincubation for 2 min at 40 °C. Chitinase activity, analyzed without the addition of any test substance, was defined as 100% activity (reference). After the initial screening of possible inhibitory effects, the cations, solvents and reagents with distinct inhibition were tested for statistical significance.

### Determination of product inhibition with N-acetylglucosamine

To determine a potential product inhibition by the chitin monomer N-acetylglucosamine, time conversion curves were recorded using 0 mM (adding distilled H_2_O) and 50 mM GlcNAc. The reaction was performed at 40 °C and pH 4.5 for 15 min. The added volume of 20 µl of GlcNAc or distilled H_2_O was subtracted from the buffer volume of 70 µl.

## Production of black soldier fly larvae (BSFL)

A total of 350,000 six-day-old BSFL (madebymade GmbH, Pegau, Germany) were weighed (pool of 30 larvae per single weight; initial weight: 7 ± 1 mg) with a precision balance (Kern & Sohn GmbH; Balingen, Germany) and randomly divided into 6 boxes (30 cm × 60 cm × 10 cm). Each box was filled with 2500 g of substrate and was offered ad libitum (0.2 g dry mass (DM)/larva). The boxes were placed in a climate chamber under controlled conditions (28 ± 1 °C, 55 ± 4% rH). Larva were fed with Binger diet (25% DM), which was composed of wheat bran, grape and carrot pomace, biscuit flour and rape presscake [29]. The analyzed crude protein (CP) and energy (GE) contents in the diet were 157 ± 4 g CP/kg DM and 19.5 ± 0.15 MJ/kg DM. At the end of the fattening period, when 40% of the prepupae were identified, 5 g of BSF exuviae were randomly selected per rearing box, separated, dried, weighed and frozen (- 20 °C).

## Hydrolysis of colloidal chitin and black soldier fly larvae

Hydrolysis of chitin was performed by incubating a 1% (w/v) colloidal chitin suspension with 0.5 and 2.0% (w/v) purified enzyme at 40 °C and optimal pH conditions under vigorous stirring. For direct hydrolysis of the chitin in the black soldier fly larvae (BSFL) 1% (w/v) of the insect exuviae was incubated with 1% (w/v) enzyme under the same conditions. The release of reducing sugars in the supernatant was detected with the dinitrosalicylic acid (DNS) assay. The DNS assay was performed according to a modified method described by Akeed et al. [30]. Samples of 200 µl were taken after defined time intervals between 0 and 24 h and inactivated at 95 °C for 15 min. Subsequently, the samples were centrifuged at 13,000 × g for 3 min to remove insoluble chitin. To 150 µl of the supernatant, DNS reagent was added in equivalent volume to a new reaction vessel and heated at 100 °C for 10 min. After cooling on ice, 200 µl of the reaction mixture was pipetted into a 96-well plate and measured at 540 nm using the Epoch 2 MicroPlate Reader. The standard N-acetyl-D-glucosamine was used for calibration. As a reference and to determine the degree of hydrolysis, colloidal chitin and BSFL were incubated with 32% hydrochloric acid for 24 h at 80 °C. The chitin suspension was neutralized with 5 M NaOH. The supernatant was measured by the DNS assay as described above.

## High-performance thin-layer chromatography (HPTLC) experiments

HPTLC experiments were performed on HPTLC glass plates Si 60 F254, 20 × 10 cm (Supelco, Merck KGaA, Darmstadt, Germany). Between 1.0 and 10.0 µl of up to 20 samples and/or standard solutions (in 50 mM sodium acetate buffer pH 4.5) were applied as 6.0 mm bands (Automatic TLC Sampler, CAMAG, Muttenz, Switzerland). The activity of the plates was adjusted by conditioning the layer with a saturated solution of magnesium chloride for 10 min at a relative humidity of less than 5% for 10 min. The plates were developed with ethyl acetate, methanol, boric acid (0.5%), glacial acetic acid in a ratio of 50:40:10:2 (v/v) after chamber saturation (20 min, with filter paper) to a distance of 70 mm from the bottom edge of the plate and then dried for 5 min (Automated Developing Chamber 2, CAMAG, Muttenz, Switzerland). Derivatization was performed by automated spraying with 3.0 ml of aniline-diphenylamine-phosphoric acid reagent (2.0 g diphenylamine, 2.0 ml aniline in 80.0 ml methanol and 10.0 ml o-phosphoric acid (85%) using a piezoelectric spraying device (Derivatizer with yellow spray nozzle at spraying level 6, CAMAG, Muttenz, Switzerland). After spraying, the plates were heated on a plate heater at 110°C for 10 min. Plate images were acquired in longwave UV (350 nm broadband) and white light after derivatization using a documentation system (TLC Visualizer, CAMAG, Muttenz, Switzerland) to detect chitin oligosaccharides and N-acetylglucosamine. Spectra were recorded from 200 to 500 nm using a scanning densitometer (TLC Scanner 3 CAMAG, Muttenz, Switzerland). Data analysis was performed using visionCATS 3.2 (CAMAG, Muttenz, Switzerland).

### Sample preparation for scanning electron microscopy

A scanning electron microscope (SEM) from Jeol GmbH (JSM-6610LV, Freising, Germany) was used to study the potential degradation of the yeast cell walls with a slightly modified sample preparation method from Ali [31]. For fixation, a 2.5% (w/v) glutardialdehyde fixation solution was prepared. A small sample of thawed cell pellets was resuspended in 2 ml of fixation solution and incubated under gentle shaking for 20 min at room temperature. The fixed cells were centrifuged at 10,000 × g for 2 min at 4 °C and resuspended in 2 ml of distilled water to remove the glutardialdehyde solution. A stepwise increase in ethanol concentration from 30 to 99.99% was chosen to dehydrate the samples. The incubation period was 7 min for each step. After incubation in 99.99% ethanol solution, the supernatant was decanted, and the cell pellet was incubated with a small amount of hexamethyldisalazane for 5 min. On an aluminum sample plate (Micro to Nano, Haarlem, Netherlands), 10 µl of the cell suspension was spread as planar as possible and dried completely. The dried samples were coated with gold atoms by cool sputtering using a Cressington Sputter Coater 108auto (Elektronen-Optik-Service GmbH, Dortmund, Germany).

### Recombinant expression of Chit36-TA in a bioreactor

Recombinant expression of Chit36-TA in *K. phaffii* BG10 was performed in a 3.5-L bench-top bioreactor (Labfors, Infors-HT, Einsbach, Germany). Transformed colonies from a YPD plate were grown in 100 ml of BMGY medium in 300 ml shake flasks. Precultures were incubated at 30 °C on a rotary shaker until an OD_600 nm_ of ~ 7 was reached. The fermentation medium used consisted of (g/l): glycerol 40, CaSO_4_ 0.93, K_2_SO_4_ 18.2, MgSO_4_*7H_2_O 14.9, KOH 4.13, H_3_PO_4_ 26.7 ml/l and Pichia trace metal (PTM_1_) solution 4.35 ml/l (g/l: CuSO_4_*5H_2_O 6.0, NaI 0.08, MnSO_4_*H_2_O 3.0, NaMO_2_*H_2_O 0.2, H_3_BO_4_ 0.02, CoCl_2_ 0.5, ZnCl_2_ 20.0, FeSO_4_*7H_2_O 65.0, biotin 0.2, and H_2_SO_4_ 5 ml/l). The pH of the fermentation basal salts medium was adjusted to 5.0 with 28% ammonium hydroxide, which served as the initial nitrogen source. The main culture was inoculated with 10% (v/v) preculture, resulting in an initial fermentation volume of 1.35 l. The pH was maintained at 5.0 using 12.5% (v/v) NH_4_OH and 1 M H_3_PO_4_. The temperature was regulated at 30 °C. The cells were grown for 18 h after inoculation until the glycerol was completely exhausted. Then, a glycerol fed batch was incubated with 50% (w/v) glycerol, (containing 12 ml of PTM_1_ trace salts per liter feed) and a feed rate of 18.15 ml/h per liter initial fermentation volume was started. After 7.5 h, the methanol fed batch (containing 12 ml of PTM_1_ trace salts per liter feed) was initiated with a stepwise methanol feed rate of 3.6 ml/h per liter initial fermentation volume for 4 h to a feed rate of 7.3 ml/h per liter initial fermentation volume for additional 88 h. In total the whole fermentation lasted 118 h and 750 ml of methanol was fed. The growth of *K. phaffii* was monitored by measuring the dry cell weight and OD_600nm_. In addition, the protein concentration and enzyme activity, measured with the 4-MUChT assay, were determined during fermentation. The supernatant was harvested by centrifugation (4,200 × g, 10 min, 4 °C), sterile filtrated (0.22 µm) and stored at - 20 °C.

### Statistical analysis

The results were subjected to statistical analysis using one-way ANOVA followed by Dunnett’s test carried out by SigmaPlot 15.0. The differences were considered statistically significant when P < 0.05 (*), P < 0.01 (**), and P < 0.001 (***). The data represent the mean of four biological replicates for the determination of the effects of cations, solvents and reagents on endochitinase activity, the mean of two biological replicates for the further experiments with the 4-MUChT assay and the mean of three replicates for all other measurements with their standard deviation (mean ± SD) using Microsoft Excel.

## GenBank accession number

The GenBank accession number for the synthetic construct gene of the endochitinase Chit36-TA originating from *Trichoderma asperellum* CBS 433.97 is OR567097*.*

## Results

### Recombinant expression of Chit36-TA in *Komagataella phaffii* in shake flasks

**Fig. 1:**
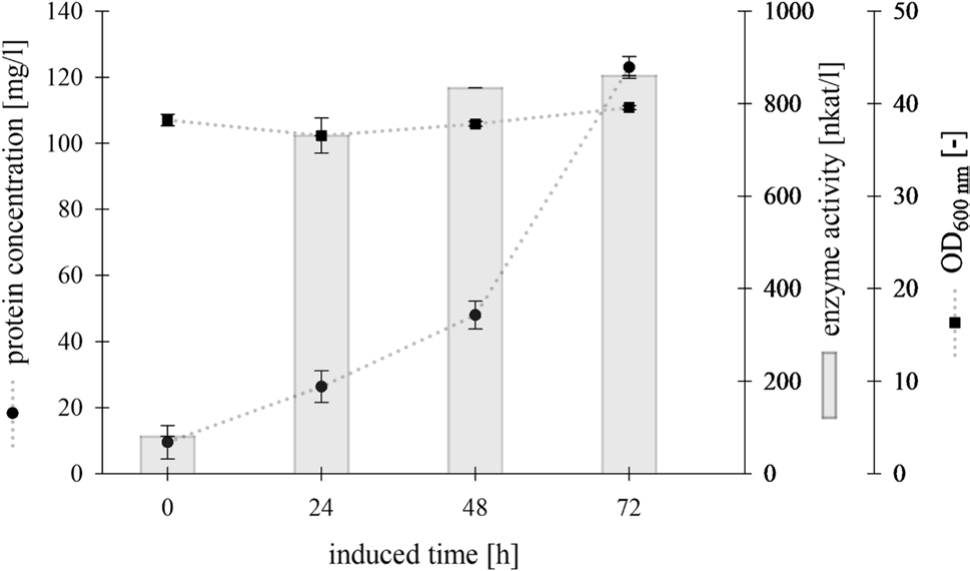
Recombinant expression of Chit36-TA from *Trichoderma asperellum* in *Komagataella phaffii* BG10 in shake flask (working volume 400 ml, BMGY medium, 28 °C, 110 rpm, induction with 0.5% methanol)

For the initial expression screening of the endochitinase Chit36-TA in *Komagataella phaffii*, the expression was performed under the control of the AOX1 promoter, utilizing a 400 ml working volume in 2 l shake flasks for up to 72 h. To monitor Chit36-TA expression, samples of the supernatant from the shake flask cultivation over 0 to 72 h were loaded on an SDS-PAGE gel for recombinant protein expression analysis (Supplementary material ESM 3, Online Resource 1), revealing a steady increase in the endochitinase band at 43 kDa over time. The observed molecular weight of 43 kDa was higher than the calculated molecular weight (36.38 kDa) possibly due to glycosylation. Furthermore, a non-distinct band on the SDS-PAGE gel was detected, which is indicative of glycosylation. A detailed time-course analysis of recombinant endochitinase expression is shown in Fig. 1. Endochitinase activity, measured with the 4-MUChT assay, increased by 75% to 724 nkat/l (43.4 U/l) during the first 24 h post induction with 0.5% (v/v) methanol, whereas between 24 and 72 h only ~ 19% increase in endochitinase activity was observed. After 72 h of induction, the activity reached a level of 861 nkat/l (51.6 U/l) and the protein concentration was 123 mg/l, corresponding to a specific activity of 7 nkat/mg.

Since chitin is a component of yeast cell walls, possible cell wall degradation of *Komagataella phaffii* by the recombinant endochitinase Chit36-TA, which may lead to the release of intracellular proteins from the expression host, was investigated by scanning electron microscopy (SEM). The surface morphologies of the recombinant *K. phaffii* cells were examined at 5000 × and 10,000 × magnification with the SEM and are presented in Supplementary material ESM 3, Online Resource 2 (A-D). No visible damage to the yeast cell walls could be detected by heterologous expression of the endochitinase compared to the empty vector control.

## Purification and deglycosylation of Chit36-TA

**Fig. 2:**
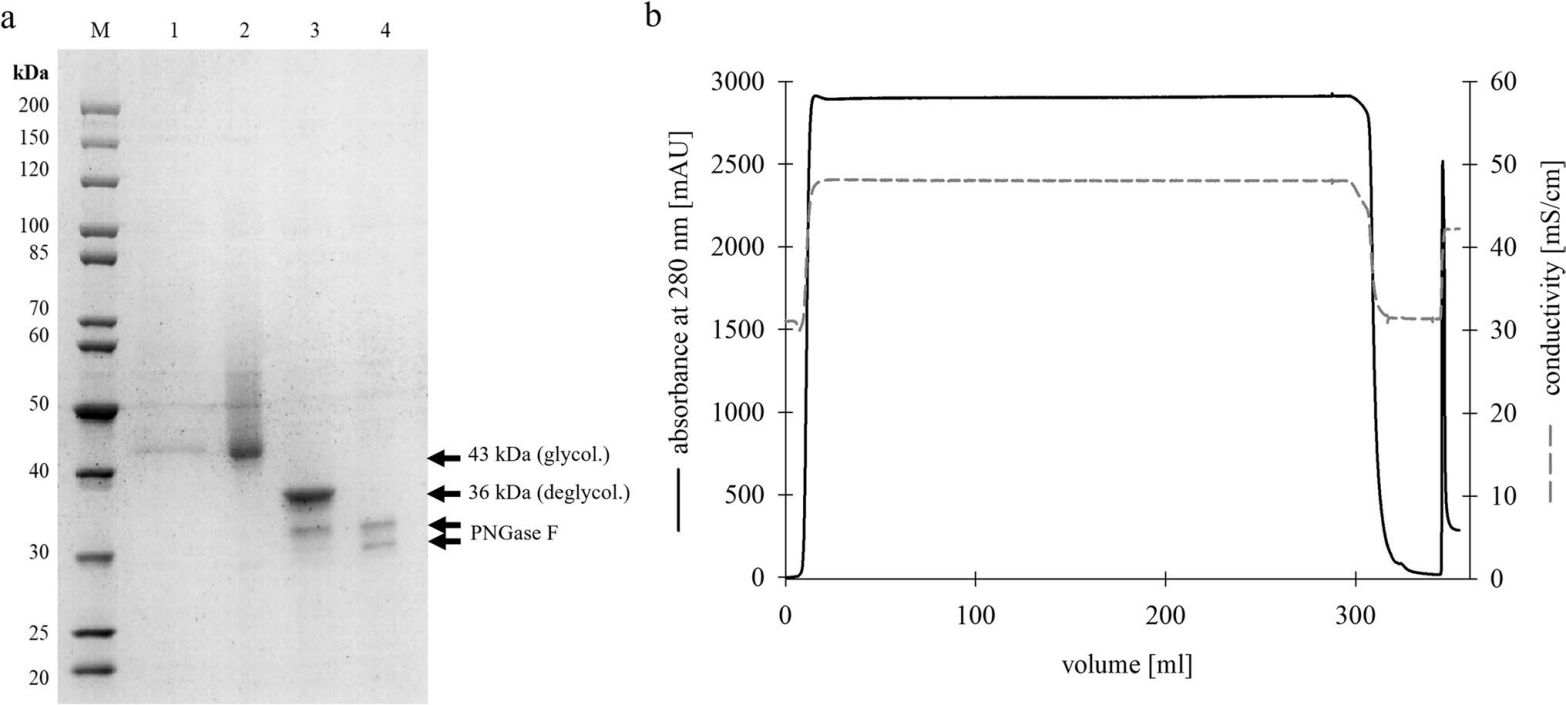
SDS-PAGE (**a**) of purified and deglycosylated endochitinase Chit36-TA samples. M: Marker PageRuler Unstained Protein Ladder; line 1: Culture supernatant after 72 h; line 2: Purified endochitinase; line 3: Deglycosylated endochitinase; line 4: PNGase F control; Ni–NTA purification chromatogram (**b**) of Chit36-TA using the protein purification system ÄKTA go with a HiTrap™ IMAC HP 5 ml column

The purification of Chit36-TA was carried out in a one-step procedure using a Ni-affinity column. Subsequent elution in a single step from 5 to 500 mM imidazole resulted in only one 43 kDa band on SDS–PAGE. To check for possible N-glycosylation, PNGase F was applied to deglycosylate Chit36-TA (Fig. 2a). Deglycosylation (Fig. 2a—lane 3) resulted in a decrease in the apparent molecular weight of the endochitinase from 43 to 36 kDa. Additionally, the previously observed non-distinct band shifted to a sharp band.

Following Ni–NTA chromatography (Fig. 2b) and subsequent dialysis, a significant increase in the protein concentration from 123 to 2755 mg/l was achieved. Moreover, the enzyme activity increased from 861 to 7847 nkat/l, resulting in an overall yield of 12%. The Ni–NTA purification led to an increase in concentration as well as a separation of further secreted proteins from the supernatant, however, due to inactivation of the enzyme the specific activity decreased during the process, which reduced the purification factor (Supplementary material ESM 3, Online Resource 3).

## Biochemical characterization of Chit36-TA

### pH-optimum, temperature maximum and temperature stability

**Fig. 3:**
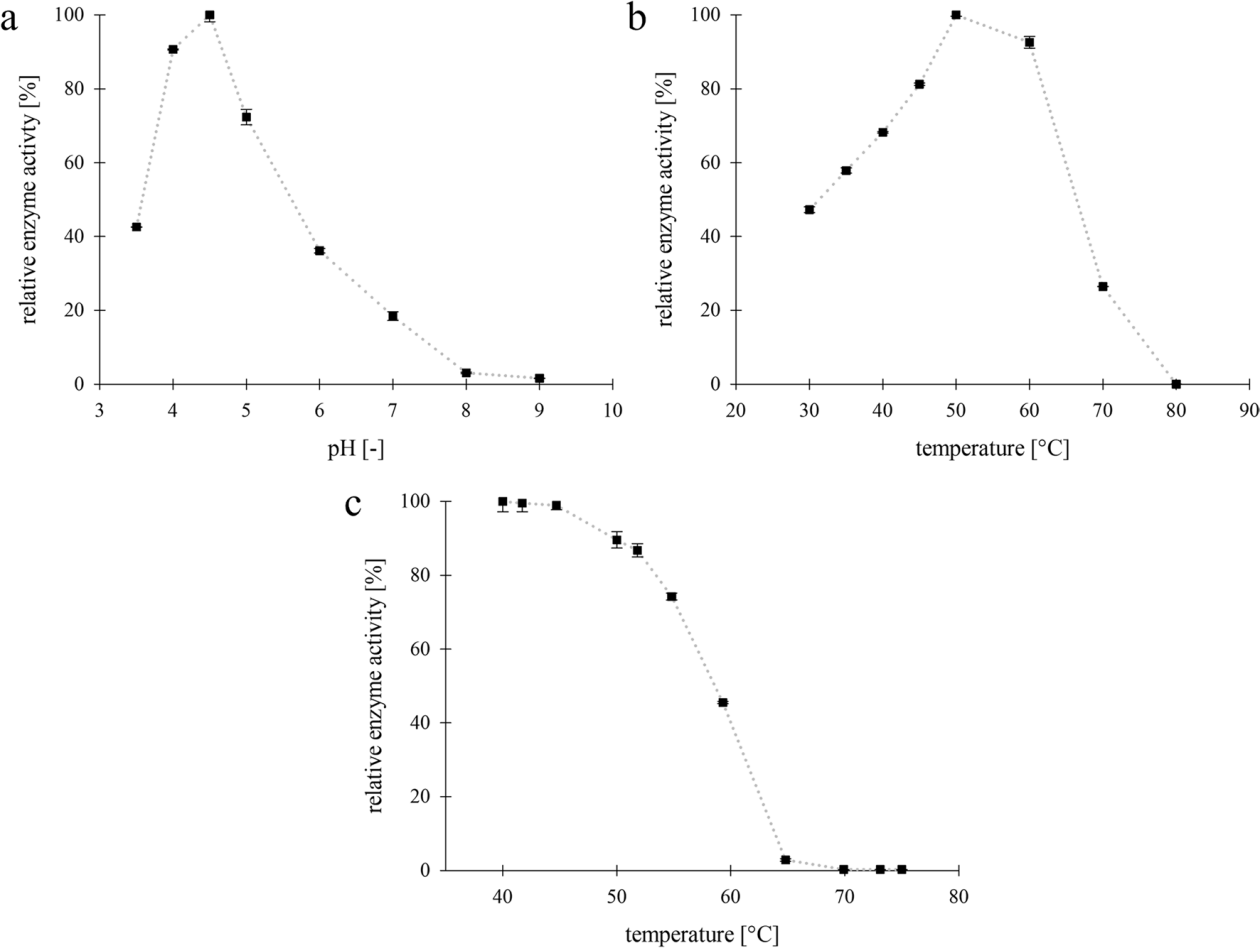
pH-Optimum (**a**) of Chit36-TA using different buffers (50 mM); Temperature maximum (**b**) of Chit36-TA determined at pH 4.5 in 50 mM sodium acetate buffer; Temperature stability (**c**) of Chit36-TA after 15 min of incubation at different temperatures

Chit36-TA exhibited the highest activity in 50 mM sodium acetate buffer at pH 4.5 and 40 °C (Fig. 3a). Chitinase activity at pH values below 4.0 and above 5.0 decreased to 43% and 36% of the maximum activity, respectively, indicating that the chitinase has a narrow pH activity range between pH 4.0 and 5.0. The optimum temperature for recombinant Chit36-TA was 50 °C, however, even at 60 °C > 93% of the maximum activity was retained. The activity declined to 26% when the temperature was increased to 70 °C (Fig. 3b). After a 15 min incubation in the temperature range from 40 to 75 °C, the chitinase was stable up to 45 °C (100% activity, Fig. 3c). With increasing incubation temperature, activity decreased rapidly. While Chit36-TA still remained 50% of its activity at 57 °C it was completely inactivated at temperatures higher than 70 °C.

### Kinetic properties

The kinetic parameters (K_m_, V_max_) were determined under standard conditions (50 mM sodium acetate, pH 4.5, 40 °C). The results were plotted and analyzed in a Michaelis–Menten diagram with substrate concentrations ranging from 5 to 70 µM 4-MUChT. V_max_ and K_m_ of Chit36-TA were calculated by nonlinear regression to be 50 nkat/mg and 289 µM, respectively. The kinetic parameters (V_max_ and K_m_) determined by Lineweaver Burk and Hanes–Woolf are in line with the nonlinear regression (Table 1).

**Table 1:** Kinetic parameters V_max_ and K_m_ of Chit36-TA by linearization according to Lineweaver Burk, Hanes-Woolf, and nonlinear regression with the substrate 4-MUChT

Substrate	Method^1^	V_max_ [nkat/mg]	K_m_ [µM]
**4-MUChT**	LB	50	293
	HW	46	298
	NLR	50	289

^1^*LB* Lineweaver Burk, *HW* Hanes-Woolf, *NLR* nonlinear regression

### Effects of solvents, reagents and cations

The possible inhibitory effects of solvents, reagents and cations were tested at different concentrations (Table 2). The addition of solvents such as ethanol, acetone, dimethyl sulfoxide (DMSO) and dimethyl formamide decreased the enzyme activity only slightly. DMSO and ethanol reduced the activity by 9 and 15%, respectively, while acetone reduced the activity by 15% to 85%. Cations such as Na^+^, K^+^, Ca^2+^, Cu^2+^, Co^2+^, Mg^2+^, Mn^2+^ and Zn^2+^ were also tested. For comparison, all the cations were determined in the form of chloride salts to exclude the influence of different anions. Metals such as sodium, potassium and magnesium had neither activating nor inhibitory effects. A slight inhibition was observed with increasing concentrations of Zn^2+^, Co^2+^ and Ca^2+^up to 10 mM. The cations Cu^2+^ and Mn^2+^ had strong negative effects. The presence of 10 mM of Mn^2+^ reduced the activity to 28%, while the addition of 10 mM of Cu^2+^ resulted in 41% of residual activity. The reagents ethylenediaminetetraacetic acid (EDTA), urea, dichlorodiphenyltrichloroethane (DDT) and *β*-mercaptoethanol did not significantly affect the activity (residual activity > 90%). Sodium dodecyl sulfate (SDS) nearly completely inactivated the chitinase at 1 mM.

**Table 2:** Effects of organic solvents, cations, reducing agents, metal chelators and denaturing agents on the activity of Chit36-TA (50 mM sodium acetate buffer, pH 4.5 and 40 °C), the standard deviation was < 5%, the significant difference refers to the 100% reference with 0 mM test substance

	Substance	Concentration[mM]	Residual enzyme activity[%]	Substance	Concentration[mM]	Residual enzyme activity[%]
Solvents	DMSO	10	91*	Acetone	10	85*
[%(v/v)]	Ethanol	10	85	DMF	10	87*
		0.1	100		0.1	91
	Na^2+^	1	99	Co^2+^	1	86*
		10	97		10	79**
		0.1	99		0.1	101
	K^2+^	1	99	Mg^2+^	1	106
Cations		10	101		10	95
		0.1	98		0.1	88
	Ca^2+^	1	90*	Mn^2+^	1	43*
		10	83**		10	28**
		0.1	90		0.1	83*
	Cu^2+^	1	72*	Zn^2+^	1	80***
		10	41**		10	93***
		0.1	84		0.1	100
	SDS	1	3*	DDT	1	100
		10	0**		10	95
		0.1	99		0.1	91**
Reagents	EDTA	1	105	β-mercaptoethanol	1	88***
		10	97		10	86***
		0.1	96			
	Urea	1	106			
		10	102			

Significant difference *P* < 0.5(*), *P *< 0.01, (**), and *P* < 0.001(***)

## Colloidal chitin hydrolysis and product inhibition

**Fig. 4:**
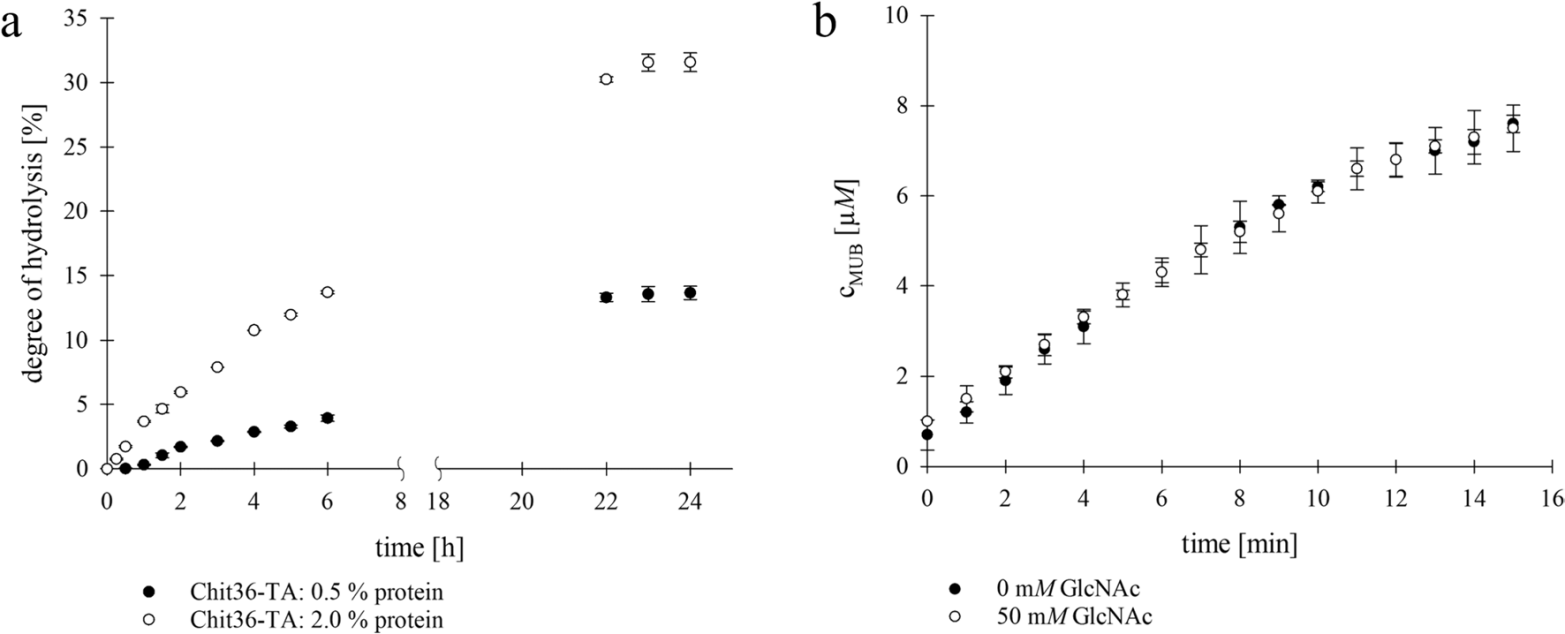
(**a**) Degree of hydrolysis (DH) of Chit36-TA with colloidal chitin (1% (w/v)) over an incubation time of 24 h (100% DH = 1.34 g/l reducing sugars); (**b**) Time-conversion curves of Chit36-TA with 0 mM and 50 mM N-acetyl-glucosamine

Hydrolysis of colloidal chitin was carried out under the optimal temperature and pH conditions for Chit36-TA (40 °C, pH 4.5) as determined above. As substrate, a 1% (w/v) suspension of colloidal chitin was used. The amount of soluble reducing sugars produced by treatment with concentrated HCl served as a reference. A maximum of 8.10 g/l of reducing sugars (N-acetylglucosamine) was measured after hydrochloric acid treatment by the DNS assay. Figure 4a shows the degree of hydrolysis (DH) increasing during incubation time. The degree of hydrolysis was defined as the percentage of reducing sugars released by Chit36-TA relative to the corresponding reference. For the hydrolysis, 0.5 or 2.0% (w/v) purified enzyme was applied. Using 0.5% (w/v) protein load a degree of hydrolysis (DH) of 14% and with 2.0% (w/v) protein load a DH of 32% were achieved after 24 h (Fig. 4a). The highest increase with initial hydrolysis rates of 0.07 (0.5% protein) and 0.24 g/l*h (0.5% protein) was observed in the first 2 to 4 h, followed by a flattening of the curve. Figure 4a illustrates the relevant phases of the colloidal chitin hydrolysis such as the initial phase, the beginning of the flattening of the curve after 6 h and the reaching of the plateau after 24 h. To explain the plateauing of the hydrolysis curves, potential product inhibition was analyzed using N-acetylglucosamine (GlcNAc).

For the product inhibition assay, a final concentration of 50 mM GlcNAc was added in the fluorometric assay and the release of 4-methylumbelliferone was analyzed over time (Fig. 4b). The time-conversion curves obtained with and without the inhibitor were almost identical. Therefore, product inhibition by N-acetylglucosamine was excluded for the endochitinase Chit36-TA.

## HPLTC analysis of the hydrolysis of colloidal chitin

**Fig. 5:**
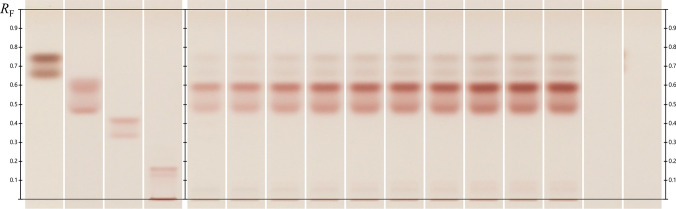
Image comparison of HPTLC fingerprints in white light (transmission) after derivatization with aniline-diphenylamine-phosphoric acid reagent; tracks 1–4: standards of N-acetyl-D-glucosamine, N,N′-diacetylchitobiose, N,N',N''-triacetylchitotriose, and N,N',N'',N''',N''''-pentaacetyl chitopentaose; tracks 5–14:obtained products after 15, 30, 60, 120, 180, 240, and 300 min, and after 22, 23, and 24 h; track 15: blank (50 mM sodium acetate buffer pH 4.5); track 16: enzyme Chit36-TA

Product analysis by high-performance thin-layer chromatography (HPTLC) using colloidal chitin was conducted to determine the hydrolytic activity of Chit36-TA. Samples were taken at intervals between 15 min and 24 h and analyzed by HPTLC for product formation. After 15 min of incubation with colloidal chitin as a substrate, Chit36-TA primarily generated N,N′-diacetylchitobiose, along with minor amounts of N-acetyl-D-glucosamine (Fig. 5). During chitin hydrolysis, an increase in N,N′-diacetylchitobiose, as well as in N-acetyl-D-glucosamine and higher chitin degradation products with a degree of polymerization greater than 5, was detected by HPTLC. This is consistent with the increase in the degree of hydrolysis shown in Fig. 4a.

## Chitin hydrolysis of black soldier fly larvae

**Fig. 6:**
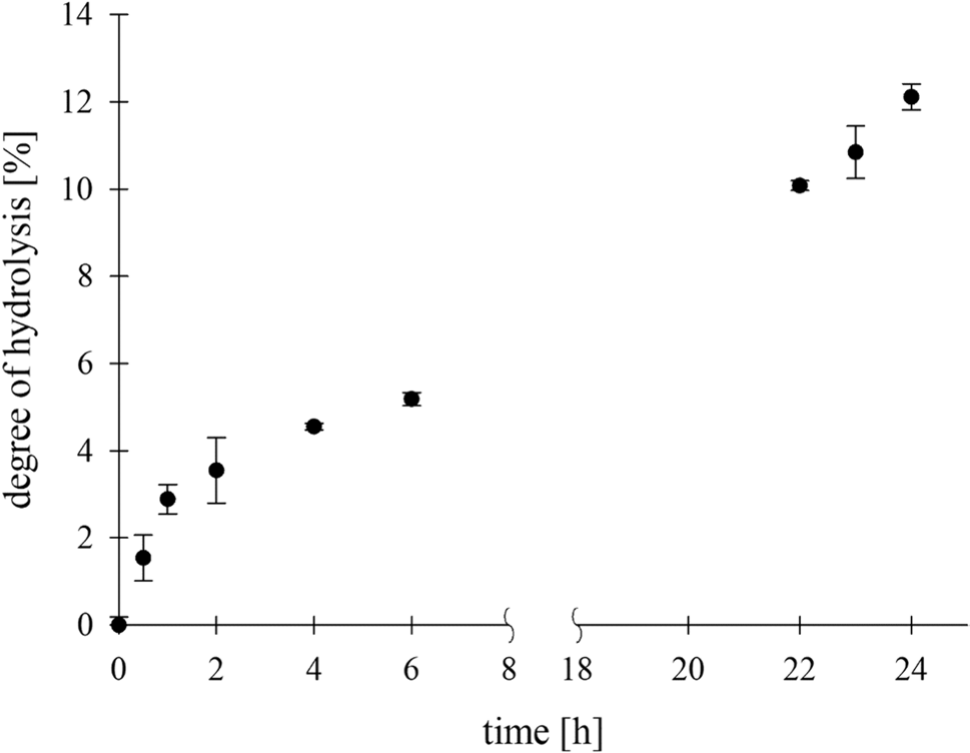
Degree of hydrolysis (DH) of Chit36-TA (1% (w/v)) with black soldier fly larvae (1% (w/v)) over incubation time of 24 h (100% DH = 1.34 g/l reducing sugars)

The exuviae of black soldier fly larvae (BSFL) were hydrolyzed under optimal temperature and pH conditions for Chit36-TA (40 °C, pH 4.5). A substrate concentration of 1% (w/v) BSFL exuviae was incubated with 1% (w/v) enzyme for 24 h. Figure 6 shows the degree of hydrolysis of chitin from BSFL with an initial hydrolysis rate of 0.04 g/l*h, whereby the curve begins to flatten out after 1 h. Chemical hydrolysis with hydrochloric acid was used as a reference, yielding a value of 1.34 g/l reducing sugars. The course of BSFL hydrolysis is similar to that of colloidal chitin except that there is no clear plateau formation and that there is still an increase in DH after 24 h. A degree of hydrolysis of 12%, corresponding to 161 mg/l of reducing sugar ends released, was achieved after 24 h.

## Bioreactor production of Chit36-TA

**Fig. 7:**
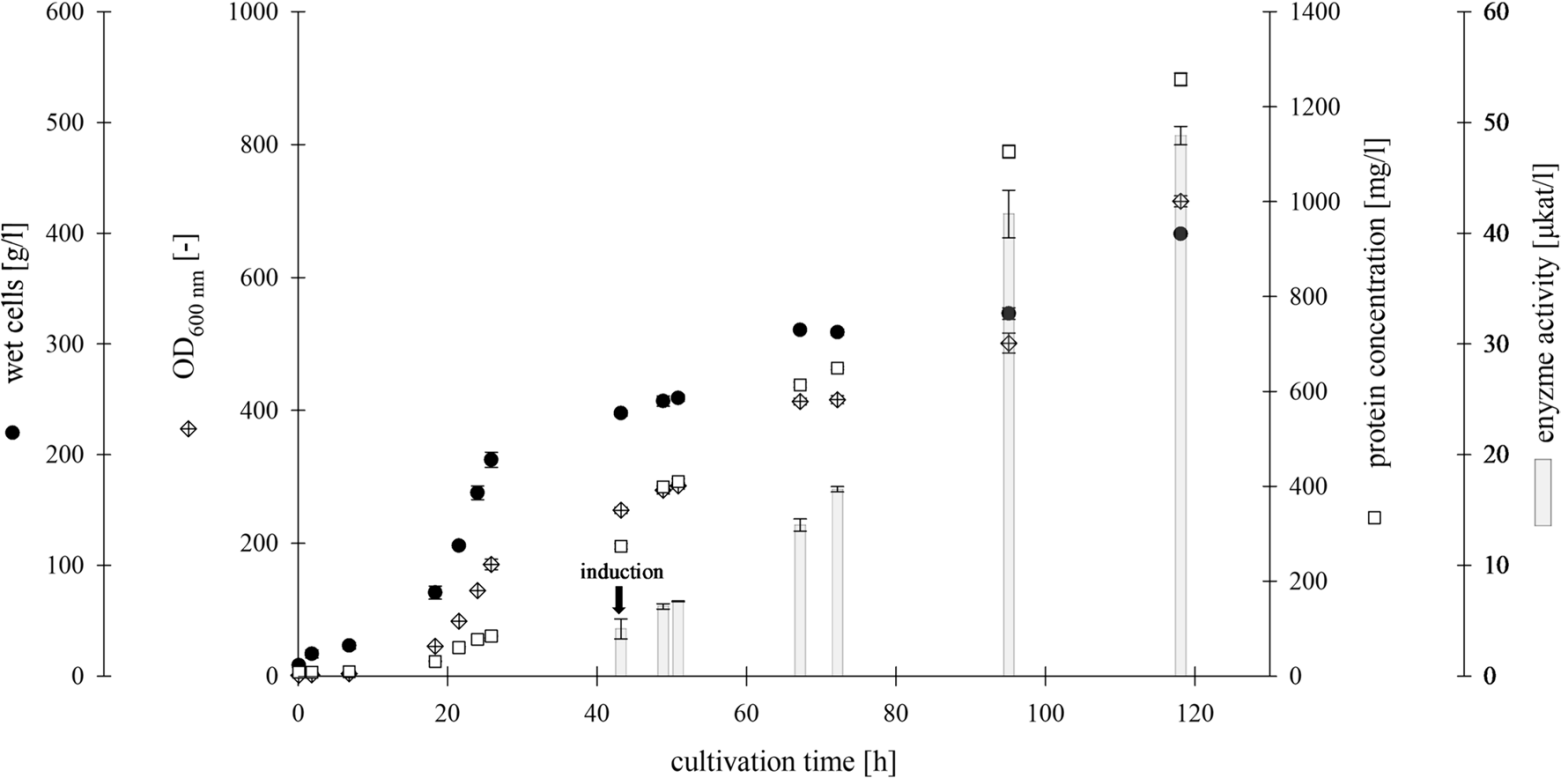
Production of Chit36-TA from *Trichoderma asperellum* in *Komagataella phaffii* BG10 (working volume 2.5 l, basal medium with PTM_1_ trace salts, 30 °C, pH 5.0, induction with methanol)

A bioreactor process was developed for the production of Chit36-TA in relevant scale, which differs from shake flask cultivation in terms of the feeding strategy, pH control and the composition of the medium. Therefore, Chit36-TA expression was transferred to a working volume of 2.5 L, yielding an OD_600 nm_ of 715 and a wet cell concentration of 399 g/l (Fig. 7). The protein concentration increased approximately tenfold to 1258 mg/l, accompanied by 55-fold higher enzyme activity compared to the shake flask cultivation. Biomass production seemed inversely proportional to protein expression, with less methanol favoring protein production. The Chit36-TA production rate peaked between 72 and 95 h, resulting in an increase in protein production from 649 to 1105 mg/l. Endochitinase activity also increased during this period, rising from 17 to 48 µkat/l (1020 to 2879 U/l), which corresponds to a space time yield of ~ 1 µkat/l/h. After ~ 120 h of cultivation, a protein concentration of 1258 mg/l and a chitinase activity of 49 µkat/l (2939 U/l) were observed.

## Discussion

In this work, a novel endochitinase was described able to efficiently hydrolyze chitin in insect larvae exuviae. The endochitinase Chit36-TA from *Trichoderma asperellum* was recombinantly expressed in *Komagataella phaffii*, biochemically characterized and used for hydrolysis of colloidal chitin as well as chitin from non-pretreated BSF exuviae.

As shown in Fig. 1, a protein concentration of 123 mg/l was reached after 72 h expression in shake flask culture. Similar protein concentrations were reached as for *Beauveria bassiana* chitinase recombinantly expressed in *K. phaffii* [32] but exceeded the achieved protein concentrations of a recombinantly expressed insect chitinase with a maximum of 5 mg/l protein [33] and a recombinantly expressed fungal chitinase with 43 mg/l [34], for which protein content was also determined by Bradford assay. Nevertheless, higher protein concentration was achieved for recombinant expression in *K. phaffii* of an endochitinase from *Trichoderma* sp*.*, reaching 365 mg/l [35]. After 72 h, a maximum endochitinase (Chit36-TA) activity of 861 nkat/l was obtained. The most significant increase to 84% of the maximum enzyme activity was observed within the first 24 h, and 97% of the total enzyme activity was obtained within 48 h after induction. A similar trend was shown by the recombinant expression of endochitinase from *Trichoderma* sp. reaching a maximum activity of 89.3 U/ml after 72 h, whereas one unit was differently defined as 1 ng of 4-MU per minute at pH 5.6 and 40 °C [35]. Compared with the endochitinase from *Trichoderma* sp*.*, the enzyme activity of Chit36-TA was ~ 100 times higher. The increase in protein concentration of 61% between 48 and 72 h (Fig. 1) without further increase in activity may be explained by other secreted cellular proteins that were also visible on the SDS PAGE (Supplementary material ESM 3, Online Resource 1). The degradation of chitin in the yeast cell wall by Chit36-TA as a reason for leakage of intracellular proteins was excluded based on the SEM results (Supplementary material ESM 3, Online Resource 2).

The production of Chit36-TA was increased by ~ 10 times to 1258 mg/l protein in the supernatant by cultivating the yeast in a 3.5-L bioreactor (Fig. 7). Compared to the heterologous expressions in *Komagataella phaffii* of endochitinase Chit33 from *Trichoderma harzianum* (630 mg/l) in a 5-L bioreactor [34] and endochitinase rChi21702 from *Sanguibacter antarcticus* (340 mg/l) in a 1-L bioreactor [36], the protein yield of Chit36-TA was ~ 2 to 4 times higher. However, there are also reports of recombinant chitinases with higher protein yields of 2.9 g/l of Chit42 from *Trichoderma harzianum* [37] and even 12.7 g/l of ChiA from *Bacillus licheniformis* [38]. Both chitinases were expressed in *K. phaffii*, whereas the heterologous expression of ChiA was additionally supported and optimized by coexpression of chaperones. After bioreactor production, a ~ 55-fold increase in enzyme activity of 49 µkat/l was achieved compared to the activity of 0.861 µkat/l for shake flask cultivation. Moreover, in comparison to the in *K. phaffii* recombinantly expressed endochitinase ech42 from *Trichoderma atroviride* (89.4 U/mg)*,* whereas one unit was defined as the amount of protein that produces 1 nmol of 4-MU per minute [39], the specific chitinase activity of Chit36-TA (38.95 nkat/mg) was 26-fold higher.

When heterologously expressed in *K. phaffii*, Chit36-TA (GenBank: OR567097) was highly glycosylated, as indicated by a non-distinct band between 43 and 60 kDa. After deglycosylation with PNGase F the SDS-PAGE showed one clear band at ~ 36 kDa (Fig. 2). The observed molecular weight of ~ 36 kDa agreed well with the calculated value of 36.38 kDa reported in UniProt (UniProt accession A0A2T3ZD30). Posttranslational modifications, e.g. glycosylations, are often observed for heterologously expressed eukaryotic genes in yeast species such as *K. phaffii*. Furthermore, as an extracellular chitinase, Chit36-TA from *T. asperellum* is most likely also originally glycosylated. The observed non-distinct band results due to the heterogeneity of glyco-sidechains, thus explaining the difference in migration on SDS gel [2].

Chit36-TA has maximum activity at pH 4.5 with a narrow range between pH 4.0 and 5.0. Other recombinant chitinases from *Trichoderma* sp. exhibited comparable pH ranges since those fungi are known to grow at slightly acidic pH. The chitinase Chit33 (X80006.1) from *Trichoderma harzianum* showed maximum activity at pH 5.0 [34] and the chitinase Ech30 from *Trichoderma atroviride* (ATCC 74058) had a pH optimum around pH 4.5–5.0 [40], which is consistent with the results for Chit36-TA (OR567097). The temperature maximum was determined at 50 °C in similar range with maximum temperatures of chitinases CHIT36 and Chit33 from *T. harzianum* being 40—53 °C [41] and 40—50 °C [34], respectively.

Chit36-TA had a maximum specific velocity of 50 nkat/mg with a comparatively low substrate affinity of 289 µM. A bacterial chitinase from *Bacillus cereus*, using the same substrate 4-MUChT or 4-MU-(GlcNAc)_3_, exhibited a K_m_ value of 50.6 µM and a V_max_ of 25 nkat/mg [42], indicating that this enzyme has a higher substrate affinity than Chit36-TA. However, the maximum specific velocity was twofold lower than that of Chit36-TA.

The inhibitory effects of several metal ions and reagents on chitinase activity were analyzed. Chit36-TA activity was slightly inhibited by higher concentrations of Ca^2+^ (10 mM) and Co^2+^ (10 mM) reducing the activity to ~ 80% and strongly inhibited by Cu^2+^ (10 mM) and Mn^2+^ (10 mM) leading to 41 and 28% residual activity, respectively. The chitinase was completely inactivated by the anionic surfactant SDS at a concentration of 1 mM. It is notable that Ca^2+^ had a slightly inhibiting effect (83% residual activity) at higher concentrations (10 mM), while other chitinases showed increased activity. For instance, an endochitinase from *Thermobifida fusca* and a chitinase rChi1602 from the marine bacterium *Microbulbifer* sp. BN3. exhibited increasing activity to 125% and 115% after the addition of Ca^2+^ [43, 44]. However, the rChi1602 also showed an activity reduction upon the addition of 10 mM of Zn^2+^ (49.2%), Cu^2+^ (60.9%), Co^2+^ (34.0%) and Mn^2+^ (75.3%) [44]. The different effects of divalent cations may be due to the different properties of metal ions in the active site of an enzyme, such as ion size, geometry, electronegativity or average coordination number, but also from the initial state of the reaction or the presence of water molecules [45, 46]. In general, Chit36-TA showed a similar tolerance to cations compared to the chitinases mentioned above.

The endochitinase Chit36-TA hydrolyzed colloidal chitin into water-soluble chitooligosaccharides (Supplementary material ESM 3, Online Resource 4). A degree of hydrolysis of 14% DH (1.1 g/l reducing sugar ends) was achieved at a final concentration of 0.5% (w/v) protein. The degree of hydrolysis increased with an increasing protein concentration (2.0% w/v) to 32% DH (Fig. 4a). The High-Performance Thin-Layer Chromatography (HPTLC) analysis revealed that the primary degradation product is diacetylchitobiose. Additionally, N-acetylglucosamine was also detected. Consequently, Chit36-TA not only exhibited endochitinase activity, which allows it to cleave chitin chains internally, but also demonstrated significant exochitinase activity, enabling it to hydrolyze colloidal chitin into di- and monomers. Furthermore, a product inhibition with N-acetylglucosamine (GlcNAc) was not detected. One reason for this could be that GlcNAc is not the main product of chitin hydrolysis of Chit36-TA. Hydrolysis products of endochitinases are often chitin oligomers with different chain lengths which may be further hydrolyzed by exochitinases such as *β*-N-acetylglucosaminidases to the monomeric sugar GlcNAc [21]. Chitinases Chi from *Bacillus licheniformis* and Chit42 from *Trichoderma harzianum* reached chitin oligomer concentrations of ~ 1.4 g/l [47] and 1.2 g/l [37], respectively, and are therefore comparable to Chit36-TA (0.5% protein). Both also utilized colloidal chitin as a substrate and incubated the hydrolysis for 24 h at 50 and 35 °C and pH 6. Furthermore, these chitinases also exhibit the capability to hydrolyze colloidal chitin into monomers, with diacetylchitobiose emerging as the primary product of the hydrolysis.

The endochitinase Chit36-TA was able to degrade chitin directly from the exuviae of black soldier fly larvae (BSFL), which is shown in Fig. 6. In comparison to the hydrochloric acid reference which liberated about 1.34 g/l, only 12% (161 mg/l reducing sugar ends) was hydrolyzed using Chit36-TA. However, in addition to chitin other components, such as proteins, are present in insect exuviae [48] that also reacts with hydrochloric acid and might interfere with the DNS assay [49]. In addition, the exuviae used from BSFL consisted of approximately 7% chitin, which corresponds to 700 mg/l chitin per 10 g/l insect exuviae. Thus, the yield of 161 mg/l enzymatically hydrolyzed N-acetylchitooligosaccharide from BSFL is the first evidence of chitin hydrolysis in insect shells. Moreover, in future applications the yield of enzymatically produced N-acetylchitooligosaccharides could be increased by pretreatment steps such as demineralization and deproteinization as in chemical chitin extraction from insects [1].

## Conclusion

In the present study, direct enzymatic chitin hydrolysis from insect exuviae has been shown for the first time by using endochitinase Chit36-TA originating from *Trichoderma asperellum* CBS 433.97 recombinantly expressed in *Komagataella phaffii*. By developing a bioreactor process, production was achieved in relevant scale for further application. Enzymatic chitin hydrolysis of insect exuviae, which are otherwise regarded as unused byproducts of the insect farming, offers an abundant resource for sustainable N-acetylglucosamine oligomer production, waste reduction and further valorization of the insect biorefinery. Further studies should therefore explore additional endo- and exochitinases to increase the efficiency and yield of chitin hydrolysis up to monomers, which can be used for various purposes in the food and cosmetic industry.

## Supplementary Information

Below is the link to the electronic supplementary material.

Supplementary file1 (Fasta 1 KB)

Supplementary file1 (GB 10 KB)

Supplementary file1 (PDF 332 KB)

## Data Availability

The authors declare that the data supporting the findings of this study are available within the paper and its Supplementary Information files. Should any raw data files be needed in another format, they are available from the corresponding author upon reasonable request.
