# Supplementary material for: Recombinant expression and characterization of the endochitinase Chit36-TA from Trichoderma asperellum in Komagataella phaffii for chitin degradation of black soldier fly exuviae
Source: Bioprocess Biosyst Eng. 2024 Aug 8;47(10):1751–66. doi: 10.1007/s00449-024-03067-4 (PMC11399303; doi:10.1007/s00449-024-03067-4)
Supplement: Supplementary file 3 — Supplementary file1 (PDF 332 KB) [file 449_2024_3067_MOESM3_ESM.pdf]

Recombinant expression and characterization of the endochitinase Chit36-TA from *Trichoderma asperellum* in *Komagataella phaffii* for chitin degradation of black soldier fly exuviae

Luisa Gebele[1] · Andreas Wilke[1] · Axel Salliou[2] · Laura Schneider[3] · Daniel Heid[1] · Tobias Stadelmann[1] · Corinna Henninger[1,5] · Uzair Ahmed[1,5] · Melanie Broszat[1] · Pascale Müller[1] · Georg Dusel[3] · Michał Krzyżaniak[4] · Katrin Ochsenreither[5] · Thomas Eisele[1]*

[1] Hochschule Offenburg, Faculty of Mechanical and Process Engineering, 77652 Offenburg, Germany

[2] École supérieure de biotechnologie de Strasbourg, 67412 Illkirch Cedex, France

[3] Technische Hochschule Bingen, Department Life Sciences and Engineering, 55411 Bingen am Rhein, Germany

[4] University of Warmia and Mazury in Olsztyn, Department of Genetics, Plant Breeding and Bioresource Engineering, Plac Łódzki 3, 10-724 Olsztyn, Poland

[5] Karlsruhe Institute of Technology (KIT), Department of Chemical and Process Engineering, 76131 Karlsruhe, Germany

***Corresponding author:** thomas.eisele@hs-offenburg.de

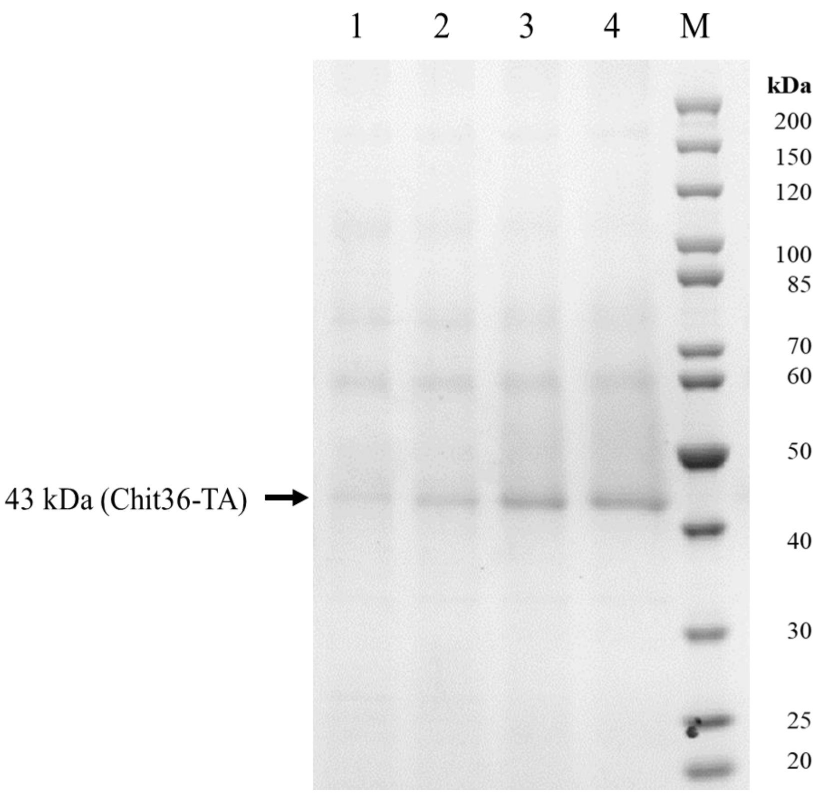

Online Resource 1 SDS-PAGE of the expression course of the glycosylated endochitinase Chit36-TA. Lines 1 to 4: Supernatant 0 – 72 h of Chit36-TA; M: PageRuler Unstained Protein Ladder as marker

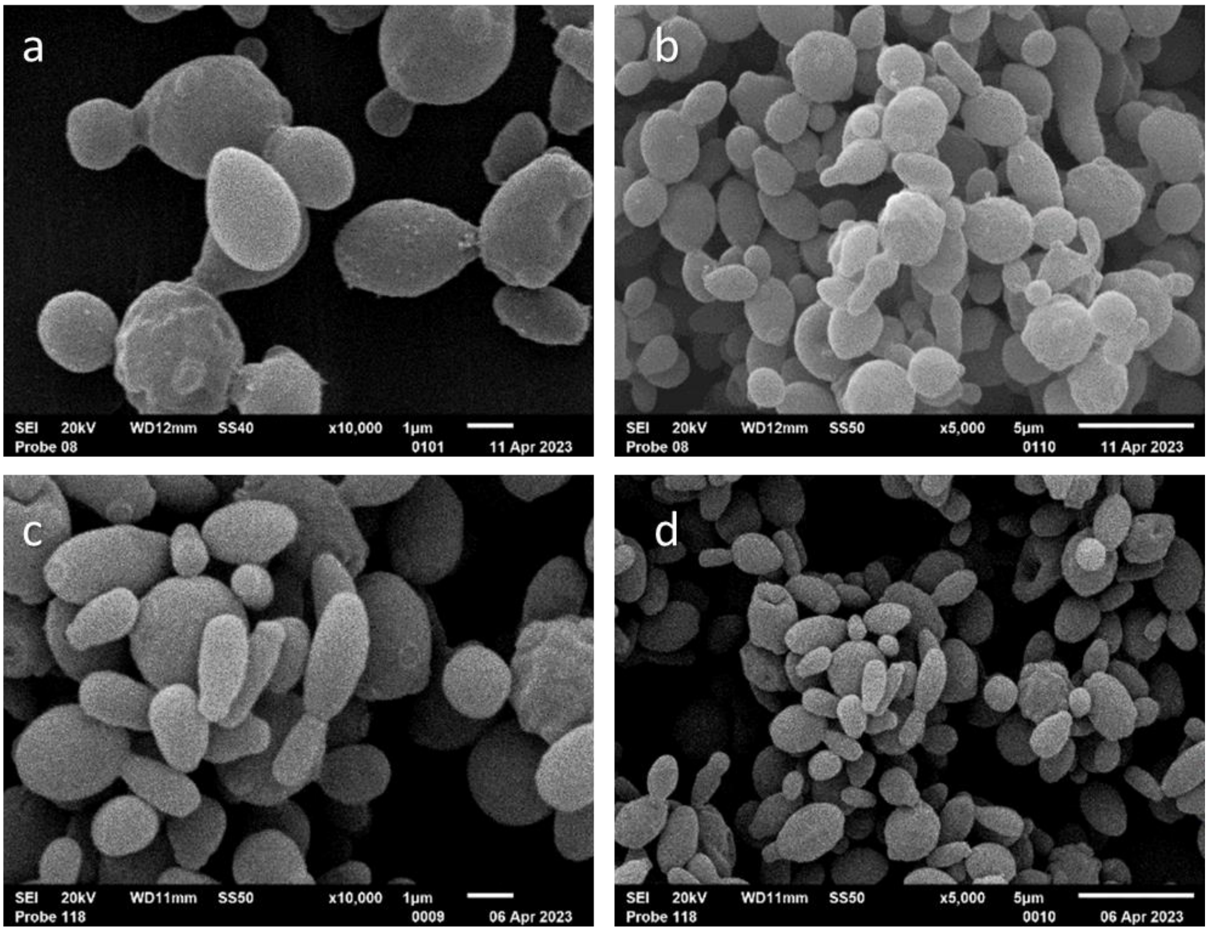

Online Resource 2 Scanning electron microscope image of the yeast cell wall of *Komagataella phaffii*. a: Negative control at 10,000x magnification; b: Negative control at 5,000x magnification; c: Chit36-TA at 10,000x magnification; d: Chit36-TA at 5,000x magnification

Online Resource 3 Purification of Chit36-TA with Ni-NTA chromatography and dialysis

**step**	**volume [ml]**	**protein concentration [mg/l]**	**total protein [mg]**	**enzyme activity [nkat/l]**	**total enzyme activity [nkat]**	**specific enzyme activity [nkat/mg]**	**yield [%]**	**purification degree [-]**
crude extract	310	123	38	860	267	7.0	100	1.0
HiTrap^{TM} IMAC HP 5ml	4	3685	15	8240	33	2.2	12	0.3
dialysis	4	2755	11	7847	31	2.8	12	0.4

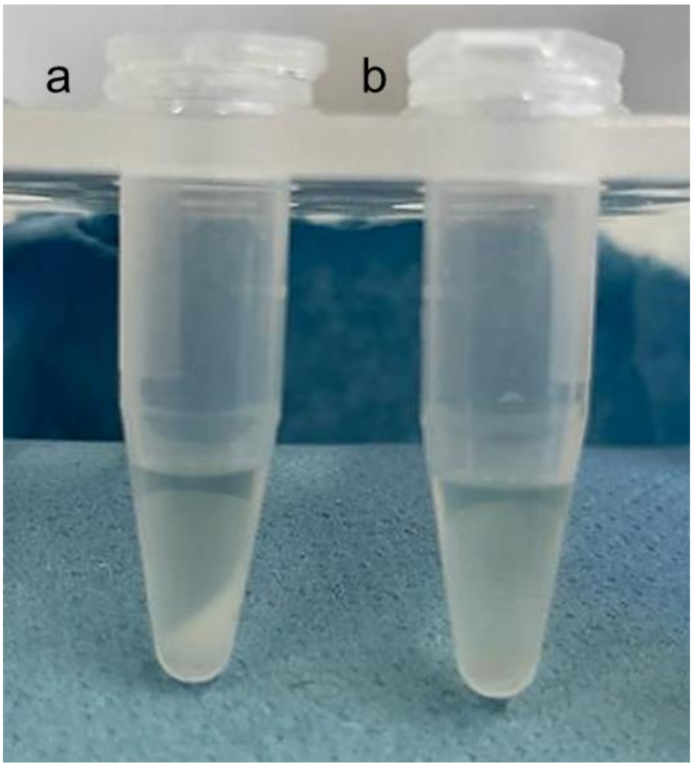

Online Resource 4 Production of water soluble chitooligosaccharides from a 1% (w/v) colloidal chitin suspension with 4% (w/v) Chit36-TA after an incubation time of 24 h. a: Centrifuged colloidal chitin after addition of inactivated (95°C, 20 min) Chit36-TA; b: Centrifuged colloidal chitin after addition of Chit36-TA
